# Current status on encephalitic alphavirus vaccines development: Advances, challenges, and global health perspectives

**DOI:** 10.1016/j.imj.2026.100249

**Published:** 2026-03-23

**Authors:** Xiaojing Yang, Yuying Ning, Chengnan Xu, Qianqian Zhang, Yangchao Dong, Yuan Wang, Fanglin Zhang, Yingfeng Lei, Wei Ye

**Affiliations:** a School of Life Sciences, Yan'an University, Yan'an 716000, China; b Department of Microbiology, Fourth Military Medical University, Xi'an 710032, China; c Medical College, Yan'an University, Yan'an 716000, China

**Keywords:** Encephalitic alphavirus, Venezuelan equine encephalitis virus, Eastern equine encephalitis virus, Western equine encephalitis virus, Vaccine

## Abstract

Alphaviruses are a globally distributed group of arboviruses responsible for two distinct disease manifestations, namely inflammatory polyarthritis and encephalitis. Among the arthritogenic viruses, chikungunya virus (CHIKV) is the most prevalent and has caused epidemics in 119 countries. Notably, two human vaccines against CHIKV have now received FDA approval. In contrast, the encephalitic alphaviruses, including Venezuelan equine encephalitis virus (VEEV), eastern equine encephalitis virus (EEEV), and western equine encephalitis virus (WEEV), are associated with substantially higher case fatality rates. Neurovirulent pathogens cause acute encephalitis in humans and animals, instigate outbreaks in endemic regions, and pose a severe public health threat. Currently, there are no licensed vaccines available for this group of viruses. However, several candidate vaccine platforms, including inactivated, live-attenuated, viral vector, and subunit vaccines, have shown promising immunogenicity and efficacy in preclinical studies, with several advancing to clinical trials. This review summarizes the current progress in vaccine development against encephalitic alphaviruses, aiming to inform future research directions.

## Highlights

• Comprehensive review on vaccine R&D for all major encephalitic alphaviruses (VEEV/EEEV/WEEV).
• In-depth comparison of vaccine platforms from inactivated to novel nanotechnologies.
• Proposes a rational roadmap for developing next-generation broad-spectrum vaccines.

## Graphical abstract

**Figure:**
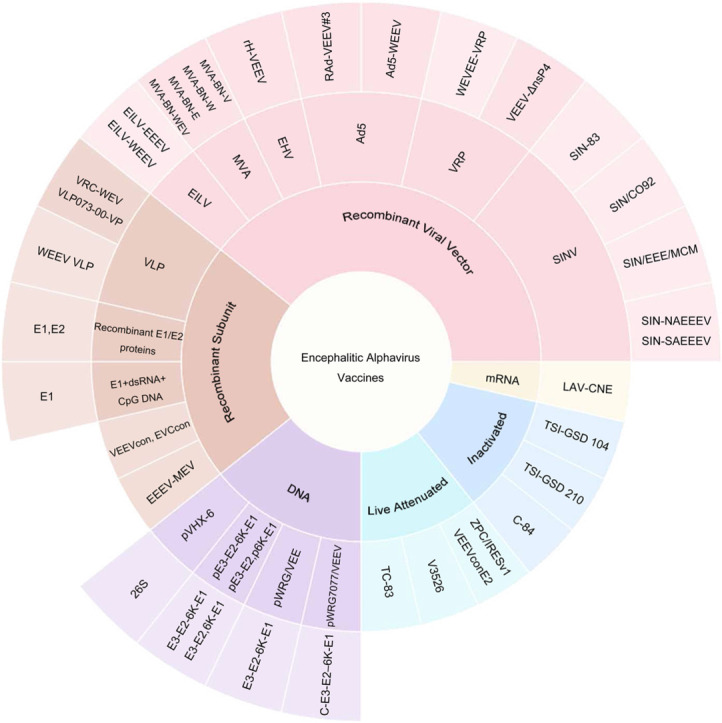

## 1 Epidemiology of encephalitic alphaviruses

Alphaviruses, belonging to the family *Togaviridae*, are classified as New World or Old World alphaviruses based on their geographical distribution. New World alphaviruses, such as Venezuelan equine encephalitis virus (VEEV), western equine encephalitis virus (WEEV), and eastern equine encephalitis virus (EEEV), are primarily found in the Americas and are the main focus of this review.^1^ They are maintained in complex transmission cycles involving mosquito vectors and vertebrate reservoir hosts, typically small mammals and birds.^2^ Human infections are incidental and occur when infected mosquitoes feed on humans. Outbreaks are often seasonal, peaking during warmer, wetter months that favor mosquito proliferation.^3^ VEEV is endemic to Central and South America, WEEV is found in western North America and parts of South America, while EEEV circulates along the Atlantic and Gulf Coasts of North America and in the Caribbean.^4^ These viruses typically cause encephalitis, whereas Old World members including chikungunya virus (CHIKV), Ross River virus, and O'nyong-nyong virus primarily induce debilitating arthralgia and rheumatoid-like arthritis.^5^ Based on this pathological distinction, alphaviruses are further classified as either arthritogenic or encephalitic. VEEV, WEEV, and EEEV are considered the primary encephalitic members.^6^

These enveloped viruses are primarily transmitted by arthropods and are characterized by a positive-sense, single-stranded RNA genome, with a diameter of approximately 70 nm and icosahedral symmetry.^6^ Infection of the central nervous system by encephalitic alphaviruses can cause meningitis and encephalitis. Although some patients recover fully, many survivors face permanent neurological sequelae such as neurasthenia, demyelination, epilepsy, chorea, paralysis, and focal deficits.^7^ The case fatality rates display a stark contrast, with VEEV approximately 1%, WEEV 3%–7%, and EEEV reaching 50%–78%.^8^^,^^9^ The clinical presentation of WEEV infection is highly age-dependent. Retrospective data indicate that the ratio of inapparent to apparent infections increases markedly with age, ranging from approximately 1∶1 in infants under 1 year, to 58∶1 in children aged 1 to 4 years, and exceeding 1000∶1 in individuals over 14 years old. Furthermore, long-term studies suggest that 15% to 30% of all encephalitis survivors experience severe neurological sequelae, with a notably higher incidence among young children. The substantial public health burden of these viruses was starkly demonstrated by outbreaks such as the 1995 VEEV event in South America, which resulted in over 75,000 human infections and more than 300 deaths.^1^

The potential for aerosol transmission, together with the relative ease of alphavirus reverse genetics, raises significant biosecurity and potential weaponization concerns. Although primarily endemic in the Americas^1^, global travel has facilitated imported cases in non-endemic regions.^2^^,^^10^^,^^11^ Consequently, increased cross-border movement heightens the risk of VEEV, WEEV, and EEEV importation.^12^ Due to their severe pathogenicity, VEEV, WEEV, and EEEV are classified as Category B priority pathogens by the National Institute of Allergy and Infectious Diseases (NIAID).^13^ As vaccination remains the most effective strategy for preventing infectious diseases, this review summarizes the current landscape of encephalitic alphavirus vaccine research to inform and guide future development strategies.

## 2 Alphavirus structure, replication cycle and antigenic targets

Alphaviruses are enveloped, positive-sense single-stranded RNA viruses belonging to the family *Togaviridae*, with a genome approximately 11–12 knt in length. Their icosahedral virions are about 70 nm in diameter and exhibit pseudo *T* = 4 symmetry.^7^ The viral genome contains two open reading frames (ORFs). The 5′ ORF is translated directly from the genomic 49S RNA to produce a polyprotein, which is cleaved into four non-structural proteins (nsP1–nsP4). These nsPs collectively form the replication complex and are responsible for viral RNA synthesis and host immune evasion. Specifically, nsP1 functions as a capping enzyme and membrane anchor for replication complexes; nsP2 is a multifunctional protease and helicase that cleaves the viral polyprotein and antagonizes the host interferon responses by suppressing JAK-STAT signaling; nsP3 scaffolds replication complex assembly and modulates host stress granule formation, while nsP4 serves as the RNA-dependent RNA polymerase (RdRp) responsible for synthesizing negative-strand genomic templates and subgenomic 26S RNAs.^14^

**Fig. 1:**
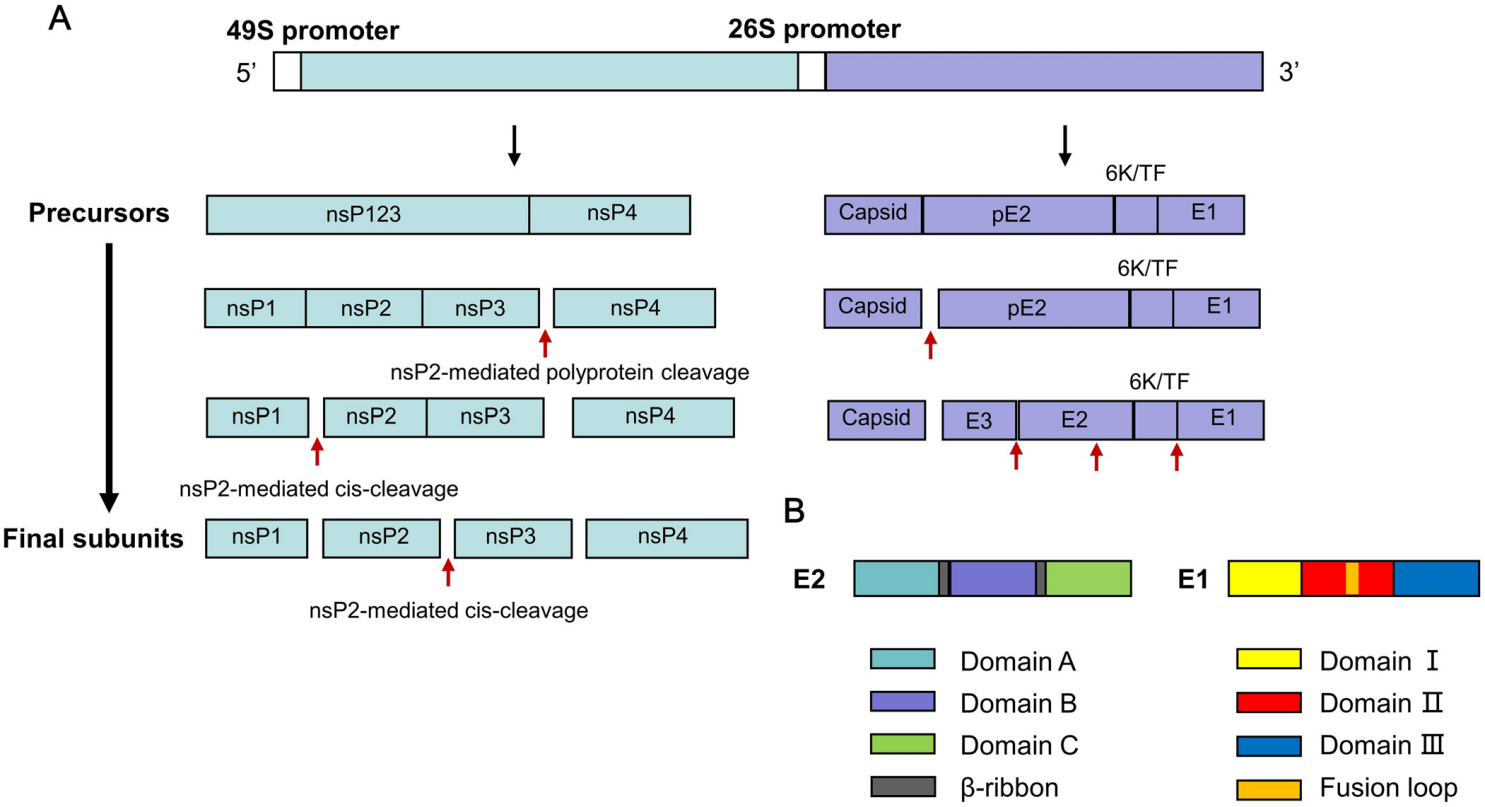
Alphavirus genome, subgenome, protein coding strategy and antigenic domain for E2 and E1. (A) Schematic of the alphavirus genomic (49S) and subgenomic (26S) RNAs. The 5′ ORF under the 49S promoter encodes the non-structural polyprotein (nsP1–4), which is processed by nsP2-mediated cleavage into four mature replicase subunits (nsP1–4). The 3′ ORF under the 26S promoter encodes the structural polyprotein (C-pE2-6K/TF-E1), which is cleaved into the capsid (C), envelope glycoprotein precursor (pE2), 6K/TF peptide, and E1 protein. pE2 is further processed into the mature E3 and E2 glycoproteins. (B) Domain architecture of the major surface glycoproteins E2 and E1, highlighting key functional regions. E2 comprises immunoglobulin-like domains A (receptor-binding site), B (fusion loop shield), and C (membrane-proximal anchor), connected by a β-ribbon. E1, a class II fusion protein, consists of domains I, II (containing the hydrophobic fusion loop), and III. *Abbrevition*: ORF, open reading frame.

**Fig. 2:**
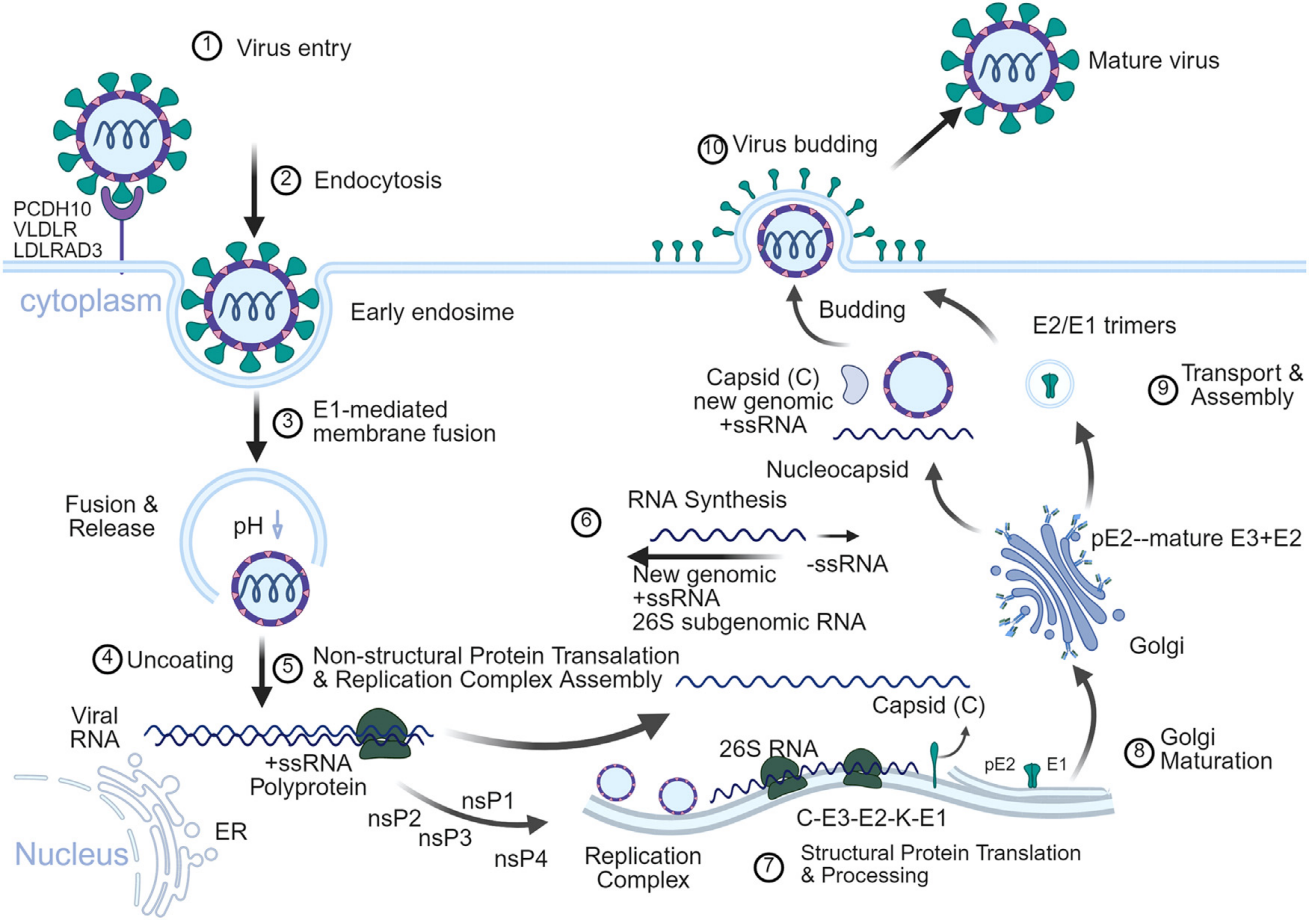
Alphavirus life cycle. The cycle begins with virus entry via receptor-mediated endocytosis. (1) Receptor binding and attachment: The viral particle’s surface spike binds to specific receptors on the host cell membrane, such as PCDH10, LDLRAD3, and VLDLR. (2) Endocytosis: The virus-receptor complex enters the cell primarily via clathrin-mediated endocytosis, forming early endosomes. (3) Membrane fusion and capsid release: When the pH decreases, it induces an irreversible conformational change in the E1 glycoprotein, thereby mediating fusion between the viral envelope and the endosomal membrane. Subsequently, the capsid is released into the cytoplasm. (4) Uncoating: The nucleocapsid dissociates, releasing the positive-strand viral genome RNA (+ssRNA). (5) Replication complex formation: The released +ssRNA directly serves as mRNA, translating into viral non-structural proteins (nsP1∶RNA capping and membrane anchoring; nsP2∶Helicase/Protease; nsP3∶Replication complex assembly; nsP4∶RdRp). (6) Negative-strand RNA synthesis and progeny RNA synthesis. (7) Structural protein synthesis and processing: The 26S subgenomic RNA is translated on the endoplasmic reticulum, producing the structural protein polyprotein precursor (C-E3-E2-6K-E1). The precursor protein is sequentially cleaved by host signal peptidases and viral proteases, such as the autocatalytic activity of protein C, generating distinct capsid protein (C), envelope glycoprotein precursor (pE2, i.e., E3 + E2), the 6 K protein, and the E1 protein. (8) The precursor is transported to the endoplasmic reticulum and processed during its transit through the Golgi apparatus. (9) Processed glycoproteins (E1 and E2) traffic to the plasma membrane. Meanwhile, newly synthesized genomic RNA is packaged by capsid proteins in the cytoplasm to form nucleocapsids. (10) Final virion assembly occurs when nucleocapsids bud through the glycoprotein-modified plasma membrane, acquiring their envelope. Created in BioRender. Wei, J. (2026) https://BioRender.com/6gepa8i.

Following replication, the 26S subgenomic RNA (approximately 4.1 knt) is transcribed at levels about threefold higher than genomic RNA. This RNA serves as the mRNA for the structural proteins and is important for both viral propagation and vaccine design. It encodes the 3′ ORF, which is translated into a structural polyprotein that is subsequently processed into the capsid (C), the glycoproteins E3, E2, E1 and the small 6 K protein^15^ (Figs. 1 and 2).

The viral life cycle begins with attachment to the host cell surface, mediated by the interaction of the E2 glycoprotein with specific cellular receptors such as protocadherin 10 (PCDH10), low density lipoprotein receptor class A domain containing 3 (LDLRAD3) and very low density lipoprotein receptor (VLDLR).^16^ Following receptor binding, the virion is internalized via clathrin-mediated endocytosis. Within the acidic environment of the endosome, low pH triggers a major conformational rearrangement in the E1/E2 spike complex. This exposes the fusion loop in domain II of the E1 protein, which inserts into the endosomal membrane, driving viral and host membrane fusion and releasing the viral nucleocapsid into the cytoplasm.^17^

For viral assembly, the structural polyprotein enters the secretory pathway. E3 acts as a chaperone for E2 folding and prevents premature E1 activation before being cleaved during maturation.^14^^,^^17^^,^^18^ The 6 K protein aids in the E1 translocation and can form a cation-selective ion channel.^19^, ^20^, ^21^ The capsid protein packages newly synthesized genomic RNA to form nucleocapsids in the cytoplasm.^22^ These nucleocapsids then bud through the plasma membrane, where they interact with the cytoplasmic tails of the E2 glycoprotein. The processed E1 and E2 glycoproteins have assembled into 80 trimeric spikes, thereby acquire their envelope to form infectious virions^17^^,^^20^ (Fig. 1). Additionally, virions can also be transported to adjacent cells via filopodial extensions enriched with E2/E1 proteins, providing a mechanism for direct cell-to-cell spread.^23^^,^^24^

Notably, this efficient 26S promoter-driven expression system is harnessed in vaccine platforms to achieve high-level antigen production. Moreover, the nsP1–4 form the backbone of self-amplifying RNA (saRNA) vaccines, in which the native structural genes are replaced with a heterologous antigen gene (e.g., SARS-CoV-2 Spike or influenza hemagglutinin). This design leverages the virus’s own amplification machinery to produce sustained, high levels of antigen from a minimal RNA dose, without generating infectious virus.^25^^,^^26^

The surface E1/E2 spikes are the key mediators of viral entry and the primary targets of neutralizing antibodies (NAbs).^12^^,^^25^ Each spike is a trimer of E1-E2 heterodimers. E1 is a class II fusion protein, with domain II containing an essential fusion loop. E2 is responsible for receptor attachment; its domain A binds host receptors (e.g., PCDH10, LDLRAD3, VLDLR), while domain B shields the E1 fusion loop at neutral pH (Fig. 2).^16^^,^^17^ During entry, receptor binding and endosomal acidification trigger conformational changes that expose the fusion loop, allowing E1 to drive membrane fusion. Given their essential roles in receptor binding (E2 domain A), membrane fusion (E1 domain II), and conformational regulation (E2 domain B), the E1 and E2 glycoproteins constitute the principal targets for vaccine-induced immunity and therefore form the basis for most current vaccine development strategies against encephalitic alphaviruses.

## 3 Research progress on encephalitic alphavirus vaccines

**Fig. 3:**
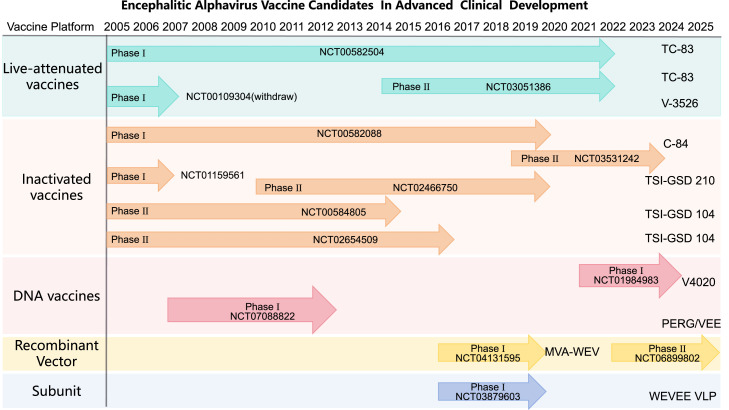
Summary of past and current encephalitic alphavirus vaccines assessed in early pre-clinical phase or clinical trials. The chart summarizes major vaccine candidates across key platforms—live-attenuated, inactivated, DNA, viral vector, and subunit/VLP vaccines—that have progressed to advanced preclinical evaluation or clinical trials. Representative clinical trial identifiers (NCT numbers) are plotted against their approximate development timeline (2005–2025), illustrating the phase of clinical assessment (Phase I–II). The figure highlights the diversity of approaches under investigation and the ongoing translational effort to advance candidates against VEEV, WEEV, and EEEV. *Abbreviations*: VEEV, Venezuelan equine encephalitis virus; EEEV, eastern equine encephalitis virus; WEEV, western equine encephalitis virus.

Currently, no vaccine has been approved for human use against encephalitic alphaviruses. Nevertheless, substantial research has yielded numerous candidates across diverse technological platforms. Many have demonstrated promising immunogenicity in preclinical studies, with a select few candidates advancing to early-phase clinical trials (Table 1). The development pipeline includes both traditional and novel strategies, including inactivated whole-virion vaccines, live-attenuated vaccines, nucleic acid-based (mRNA/DNA), viral vectored, and recombinant subunit vaccines (Fig. 3, Table 2). Although the recent FDA approval of two CHIKV vaccines marks a milestone for arthritogenic alphaviruses, vaccine development against encephalitic members like EEEV and VEEV remains largely at the preclinical studies, primarily evaluated in mouse and non-human primate (NHP) models. Two major hurdles persist in this field: achieving broad cross-protection against antigenically distinct viruses such as EEEV and VEEV, and ensuring durable immunity without compromising safety. The following section details the current status of these vaccine candidates, organized by platform technology and developmental stage.

**Table 1:** Characteristics of available vaccines against encephalitic alphaviruses.

Vaccine platform	Vaccine name	Target virus	Key antigen/gene construct	Animal model	Vaccine immunogenicity Efficacy outcomes	Immunization regimen	Advanced to clinical stage	References
Live-attenuated	TC-83	VEEV	E2-6K-E1 (TrD I/AB)	Male Swiss ICR mice, 21 to 24 days or 6 week-old	The relatively lower rate of antibody conversion	Single dose (5 to 200 PFU, i.p.)	Phase Ⅱ	^27^^,^^28^
	V3526	VEEV	E2-6K-E1 (TrD I/AB)	Mixed-breed horses (3 to 14 year-old)	Serum neutralizing antibody titers rose significantly, with a GMT of 1∶316	Single dose (10²–10⁷ PFU, s.c.)	Phase I	^35^^,^^36^
	ZPC/IRESv1, VEEVconE2	VEEV	E2 (ZPC-738)	Female CD-1 mice, 8 week-old	The vaccine provided broad protection, including partial cross-protection against MADV	Single dose (10⁵ PFU, s.c.)	No	^34^
	V3526 RdRp	VEEV	E2-6K-E1 (TrD I/AB)	Female CD-1 mice (6 to 8 week-old )	It reduced tissue tropism while maintaining protection and immunogenicity in mice.	Single dose (10⁴–10⁵ PFU, s.c.)	No	^37^
Inactivated vaccines	C-84	VEEV	E2-6K-E1 (TrD I/AB)	Healthy adults aged 18 and above	High-titer, broadly cross-reactive, and durable (≥ 14 months) neutralizing antibodies.	3 doses (0.5 mL, s.c.; 0.1 mL yitongyi, i.d.)	Phase Ⅱ	^38^^,^^39^
	TSI-GSD 210	WEEV	E2-6K-E1 (CM-4884)	Healthy adults aged 18 and above	The immunogenicity varied between lots, but the vaccine provided sustained protection.	3 doses (0.5 mL/dose, s.c.)	Phase Ⅱ	^40^
	TSI-GSD 104	EEEV	E2-6K-E1 (PE-6)	At-risk laboratory workers aged 18–65 years	Primary series: 84%; long-term: 75% (PRNT80 ≥ 40).	3 doses (0.5 mL/dose, s.c.); 3 doses (0.1 mL/dose, i.d.)	Phase Ⅱ	^41^
Nucleic acid	V4020	VEEV	E2-6K-E1 (TrD I/AB)	Female BALB/c mice (4 to 8 week-old)	No adverse reactions and high levels of neutralizing antibodies were produced	Single dose (50 μL, s.c./i.m.)	Phase I	^51^
	pVHX-6	WEEV	26S (71V-1658)	Female BALB/c mice, 17–25 g	Complete homologous protection; partial heterologous protection (Fleming 62%, CBA87 50%)	4 doses (5 μg/dose, gene gun, i.m.)	No	^43^
	pE3-E2-6K-E1	WEEV	E3-E2-6K-E1	Female BALB/c mice, 17–25 g	Complete homologous protection; partial heterologous protection	3 doses (2 μg/dose, gene gun, i.m.)	No	^44^
	pWRG/VEE	VEEV	E3-E2-6K-E1 (TrD I/AB)	Female BALB/c mice (6 to 8 week-old) Female New Zealand White rabbits (3 to 3.5 kg); Healthy adult male cynomolgus macaques (≥ 5 kg)	Demonstrated strong immunogenicity and complete protection in three animal models.	Single dose (20 μL/Mouse, 0.5 mL/NHP/Rabbit, i.m. EP)	Phase I	^48^
Recombinant viral vector	SIN-83	VEEV	C-E2-E1 (TC-83)	Weanling NIH Swiss mice	Lower neutralizing antibody titer than TC-83, but ensures complete protection with significantly reduced residual virulence.	Single dose (10³–10⁶ PFU, s.c.)	No	^61^
	SIN/EEE/McM	EEEV/WEEV	C-E3-E2-6K-E1	Female and pregnant NIH Swiss mice (6 to 8 week-old)	SIN/EEE/McM provides complete protection with strong immunogenicity and high safety.	Single dose (10^3.5^–10^5.8^ PFU, s.c.)	No	^62^
	SIN—NAEEEV	EEEV	C-E3-E2-6K-E1	Cynomolgus macaque weighing 3–6 kg	Vaccine provided 82% protection against lethal aerosol challenge in macaques.	Single dose (10⁵ PFU, s.c.)	No	^63^
	MVA-BN series	V/W/EEV	E3-E2-6K-E1	Female BALB/c mice (16–18 g)	Vaccines achieved 100% protection against lethal alphavirus aerosol challenge in mice	2 doses (10⁸ TCID₅₀/dose, s.c.)	Phase Ⅱ	^70^
Subunit	WEVEE VLP	W/E/VEEV	C-E3-E2-6K-E1	Healthy adults aged 18–50 years	Vaccine showed promising safety and immunogenicity, inducing neutralizing antibodies against all three target viruses.	2 doses (5–60 μg/dose, i.m.)	Phase I	^84^
	Recombinant E1/E2	WEEV	E1/E2 (71V-1658)	Female BALB/c mice (6 to 8 weeks old)	Protective immunity was induced in some mice.	4 doses (50 μg /dose, i.p.)	No	^82^^,^^83^
	Baculovirus lysates	VEEV	C–E3–E2–6K–E1	Female BALB/c mice (6 to 8 weeks old)	No adverse reactions; high levels of neutralizing antibodies were produced	2 doses (15 μg, i.p.)	No	^85^

*Abbreviations*: s.c., subcutaneous; i.m., intramuscular; i.p., intraperitoneal; i.d., intradermal; VEEV, Venezuelan equine encephalitis virus; TrD, Trinidad donkey; RdRp, RNA-dependent RNA polymerase; WEEV, western equine encephalitis virus; EEEV, eastern equine encephalitis virus; PRNT80, plaque reduction neutralization test; ICR, Institute for Cancer Research; GMT, geometric mean titer; MADV, madariaga virus; EP, electroporation; NIH, national institutes of health.

**Table 2:** Encephalitic alphavirus vaccine candidates in advanced clinical development.

Vaccine platform	Vaccine	Clinical trail
Live-attenuated	TC-83	NCT00582504, NCT03051386
	V3526	NCT00109304 (withdraw)
Inactivated	C-84	NCT00582088, NCT03531242
	TSI-GSD 210	NCT01159561, NCT02466750
	TSI-GSD 104	NCT00584805, NCT02654509
DNA	V4020	NCT07088822
	PERG/VEE	NCT01984983
Recombinant Vector	MVA-WEV	NCT04131595, NCT06899802
Subunit	WEVEE VLP	NCT03879603

### 3.1 Live attenuated vaccines

Live attenuated vaccines (LAVs) aim to retain the immunogenicity of the wild-type virus while significantly reducing its virulence. A foundational LAV against encephalitic alphaviruses is the TC-83 vaccine, developed by the U.S. Army Medical Research Institute of Infectious Diseases (USAMRIID). This candidate was derived from the virulent VEEV Trinidad Donkey (TrD) strain through 83 serial passages in guinea pig heart cell culture. This process introduced attenuating mutations across the viral genome, including the 5′ untranslated region (UTR), nsP3, E2, E1, and the 3′ UTR.^27^^,^^28^ While TC-83 induces durable, protective immunity in horses and remains the only vaccine approved for at-risk laboratory and military personnel in the USA, its application is limited by adverse reactions. Approximately 20% of vaccinees experience adverse effects like viremia, fever, or leukopenia. Persistent concerns regarding potential reversion to virulence and waning antibody titers have thus far precluded its broader public licensure.^29^, ^30^, ^31^

Building upon the TC-83 benchmark, researchers have engineered novel LAVs candidates with enhanced safety profiles. Read et al.^32^ developed two such vaccines: ZPC/IRESv1, which replaces the VEEV subgenomic promoter with an encephalomyocarditis virus internal ribosome entry site (IRES) element, and VECCconE2, which incorporates a consensus sequence for the E2. In mouse challenge models, a single subcutaneous dose of 10^5^ PFU of either candidate provided complete protection against multiple VEEV strains and even elicited partial cross-protection against an EEEV strain.

A second major approach involves rational attenuation through reverse genetics. USAMRIID and the University of North Carolina developed V3526 from a V3000 cDNA clone by introducing a lethal mutation at the E2 furin cleavage site alongside a compensatory mutation at E1 residue 253.^33^ Extensive preclinical evaluation has demonstrated that V3526 confers robust protection against VEEV challenge in both rodent and NHP models.^34^^,^^35^ Its immunogenicity extends to large animals; a study by Fine et al.^36^ showed that doses as low as 10^2^ PFU elicited NAbs in 24 of 25 horses within 14 days, effectively protecting them against challenge with the virulent TrD strain without adverse events.

To further improve safety, subsequent research has focused on introducing additional attenuating mutations. Haines et al.^37^ generated V3526 variants with targeted mutations in the RdRp. A single immunization with these RdRp mutants provided complete protection and robust antibody responses in mice, suggesting a potentially superior safety profile compared to the original V3526 vaccine.

### 3.2 Inactivated vaccines

Inactivated vaccines represent a conventional strategy for encephalitic alphaviruses, though their development faces consistent challenges. The C-84 vaccine, a formalin-inactivated derivative of the live-attenuated TC-83 strain, was developed by USAMRIID primarily as a booster for individuals who respond inadequately to the initial TC-83 vaccination. Although two clinical trials (NCT03531242, NCT00582088) were initiated to evaluate its safety, immunogenicity, and boosting schedule, the final results remain pending. Consequently, C-84 remains available only for emergency use among high-risk personnel under an Investigational New Drug (IND) protocol.^38^^,^^39^

For WEEV, the candidate TSI-GSD 210 is derived from the attenuated CM-4884 strain grown in primary chicken embryo fibroblasts (CEFs). Long-term clinical observations spanning 24 years revealed considerable variability in immunogenicity across different production lots. For instance, the Plaque Reduction Neutralization Test (PRNT80) response rates declined from 42% (326/770) in the late 1980s to 16% (14/87) in later years, although one specific lot (3-1-92) achieved a substantially higher rate of 89% (17/19).^40^ These inconsistencies, along with the complexity and variability of primary CEF-based manufacturing, have ultimately hindered commercial scale-up and licensure.

A parallel situation exists for EEEV with the TSI-GSD 104 vaccine, produced from the PE-6 strain cultured in CEFs. A Phase II trial (NCT00584805) demonstrated that a two-dose primary series induced protective NAbs titers in 60% of subjects, increasing to 84% following a six months booster.^41^ Despite a long history of safe use under IND protocols, the constraints of cell culture-based production have similarly hindered its path to broader availability.

To address the need for a unified solution against multiple threats, Lam et al.^42^ developed a novel trivalent inactivated vaccine targeting VEEV, WEEV, and EEEV. This candidate employs a chimeric Sindbis virus backbone expressing the structural proteins of the target viruses, which is subsequently inactivated with hydrogen peroxide (H_2_O_2_) to enhance safety while preserving key conformational epitopes. In animal models, a two-dose regimen provided complete protection to mice against lethal aerosol challenge with all three viruses and elicited robust, balanced NAb responses in NHPs.

Collectively, the development of inactivated vaccines for encephalitic alphaviruses is constrained by several common challenges. These include the frequent need for booster doses to sustain immunity, often suboptimal long-term immunogenicity, and complex manufacturing processes. These shared limitations underscore the necessity to develop next-generation vaccine platforms capable of providing durable protection through more streamlined production pathways.^39^, ^40^, ^41^

### 3.3 Nucleic acid vaccines

#### 3.3.1 DNA vaccines

DNA vaccines represent an investigational platform for immunization against encephalitic alphaviruses. Early studies by Nagata et al.^43^ focused on a DNA vaccine (pVHX-6) encoding the WEEV 26S genes. While four doses administered via gene gun achieved 100% protection in mice against the homologous WEEV strain 71V-1658, efficacy significantly declined against heterologous strains Fleming and CBA87 (62% and 50% survival, respectively). Subsequent work by Gauci et al.^44^ identified that WEEV DNA constructs expressing the full E3-E2-6K-E1 polyprotein or the 6K-E1 unit conferred complete homologous and partial heterologous protection, whereas the E3-E2 was ineffective. A key finding from these studies was that the omission of the capsid protein, which can modulate host immune responses, likely enhanced protection by minimizing viral immune evasion.^45^ This capsid-deletion strategy was later validated by Dupuy et al.^46^, who demonstrated that a capsid-less WEEV DNA vaccine delivered via electroporation offered complete protection against aerosol challenge with the heterologous CBA87 strain.

Similarly, DNA vaccine efforts against VEEV have shown progress. Riemenschneider et al.^47^ developed a candidate encoding the VEEV I/AB structural proteins, which protected mice from subcutaneous challenge. Building on this, Dupuy et al.^48^ engineered pWRG/VEE, which expresses the VEEV E3-E2-6K-E1 genes. Intramuscular electroporation (IM-EP) delivery of this vaccine induced high-titer NAbs in mice, rabbits, and cynomolgus macaques, and protected NHPs against aerosol VEEV challenge. This vaccine advanced to a Phase I clinical trial (NCT01984983), which confirmed the safety, tolerability, and sustained immunogenicity of both intramuscular and intradermal electroporation over one year.^49^ Subsequent work has explored needle-free jet injection as an alternative delivery method for primates.^50^

A significant recent advancement is the V4020 vaccine, which incorporates multiple engineered features for enhanced safety and stability. These include gene rearrangement, known TC-83 attenuating mutations, and synonymous codon substitutions.^51^ A single subcutaneous dose protected mice from VEEV challenge without adverse effects and demonstrated significantly reduced neuroinvasiveness compared to the TC-83 vaccine.^52^ Manufacturing innovation also complements this design; novel production methods using PCR-amplified fragments (TAP) to generate the V4020 virus in Chinese hamster ovary (CHO) cells offer a plasmid-free manufacturing pathway.^52^, ^53^, ^54^ Beyond structural protein targets, Bounds et al.^55^ explored a multi-epitope DNA vaccine (pWRG7077/VEEV) focusing on HLA-II T-cell epitopes. Although IM-EP provided only 20% protection against challenge, the study established a proof-of-concept for T-cell-focused vaccine strategies.

Despite these extensive preclinical efforts and limited clinical progress, no DNA vaccine against an encephalitic alphavirus has achieved licensure. Key scientific hurdles include inconsistent cross-strain protection and the need for more robust immunogenicity. Furthermore, the requirement for high-containment (BSL-3/4) facilities to work with these viruses, combined with their low incidence, has further constrained commercial investment and momentum needed to advance these candidates through large-scale clinical trials.^43^^,^^46^^,^^55^

#### 3.3.2 mRNA vaccines

The unparalleled achievements of mRNA vaccines against SARS-CoV-2 highlight the potential of this platform, but its application in encephalitic alphaviruses remains in its early stages.^56^ Major barriers include the high pathogenicity of these viruses, their focal geographical distribution, and the stringent biosafety containment required for research, all of which have deterred extensive investment.^53^ In terms of delivery systems, lipid nanoparticles (LNPs) have been proven to effectively deliver mRNA and elicit a robust immune response, thus providing a reliable vector for the development of encephalitis. Validated through billions of doses, LNP delivery systems can directly encapsulate antigen-encoding mRNA, surmounting traditional delivery bottlenecks and enabling rapid preclinical proof-of-concept. In antigen design, engineering modifications to viral envelope proteins can be achieved by using pre-fusion stabilisation strategies to target pathogens such as coronaviruses—a precise upgrade over traditional inactivated vaccines. Furthermore, the inherent flexibility of the mRNA platform, particularly the self-amplifying mRNA platform, has been fully leveraged, enabling the induction of potent and durable immune responses at very low doses, making it an ideal rapid-response vaccine platform for addressing potential biological threats.^57^, ^58^, ^59^ However, for encephalitic alphaviruses specifically, the field faces distinct challenges despite progress in related areas such as flaviviruses. Key challenges include biosafety concerns, which are being addressed by AI-driven antigen design and optimized LNP delivery for precise control, and thermal stability in tropical regions, where novel carriers (such as freeze-dried LNPs and engineered extracellular vesicles) show promise to overcome cold chain limitations. Moreover, shifting from a reactive approach to proactive development—supported by universal “prototype pathogen” platforms and immunoinformatics tools is pivotal to accelerating future candidate vaccines against these under-researched viruses.

A leading approach involves a saRNA vaccine encoding the structural proteins of the live-attenuated VEEV TC-83 strain. Formulated in a cationic nanoemulsion (CNE), this candidate, designated LAV-CNE, and demonstrated an improved safety profile while eliciting immune responses comparable to the TC-83 virus. Crucially, it provided complete protection in mice against lethal aerosol challenge with virulent VEEV, establishing proof-of-concept that a saRNA vaccine can recapitulate the robust efficacy of a live-attenuated platform while mitigating its safety risks.^60^ However, key challenges remain. The ectopic expression of structural genes from highly virulent strains poses biosafety concerns during development, and ensuring thermostability for deployment in endemic tropical regions is a critical hurdle. The absence of substantial follow-up work highlights the current niche status of encephalitic alphaviruses within the rapidly advancing field of mRNA vaccinology.

### 3.4 Recombinant vector vaccines

Recombinant vector vaccines utilize engineered, non-pathogenic or attenuated viruses as delivery platforms to express protective antigen genes from encephalitic alphaviruses such as VEEV, WEEV, and EEEV. By leveraging the natural infection and gene expression machinery of these viral vectors, such vaccines efficiently stimulate humoral and cellular immune responses, often exhibiting self-adjuvanting properties.^58^

#### 3.4.1 Chimeric vaccines based on Sindbis virus vector

Sindbis virus (SINV), an alphavirus that is rarely pathogenic in humans, serves as a versatile vector platform. Its utility stems from the ability to replace its own structural gene region (C-E3-E2-6K-E1) with corresponding genes from a target pathogen, creating a replication-competent chimera driven by the SINV non-structural proteins (nsP1–4). A well-established reverse genetics system allows for rapid chimera construction. This platform supports efficient transduction of a variety of cell types and facilitates high-level antigen expression. While its packaging capacity imposes a size constraint, typically limiting foreign genetic inserts to fewer than 3.2 kb, this is generally adequate to accommodate the structural cassettes of other alphaviruses.

Multiple chimeric vaccines have been developed on this SINV backbone. Paessler et al.^61^ developed SIN-83, a recombinant SINV expressing the structural proteins of VEEV TC-83. This candidate was non-pathogenic in mice yet elicited robust immunity, characterized by strong NAbs and T-cell activation. Atasheva et al.^62^ constructed a panel of chimeras, including SIN/CO92, SIN/EEE/McM, and SIN/SIN/McM, by inserting the structural genes from WEEV and EEEV strains into the SINV AR339 backbone. Immunization with these chimeras conferred complete protection in mice against lethal intranasal WEEV challenge, with survival rates strongly correlating with high NAbs titers. Roy et al.^62^^,^^63^ developed SIN—NAEEEV, which expresses the structural proteins of a North American EEEV strain. In a stringent cynomolgus macaque model, a single aerosol dose of this vaccine resulted in an 82% survival rate following aerosol EEEV challenge, significantly outperforming a chimera based on a South American EEEV strain.

The SINV vector platform offers several distinct advantages for vaccine development. It enables rapid development and can be manufactured at relatively low cost. Most importantly, the low seroprevalence of SINV in human populations and the absence of its structural proteins in the final vaccine chimera help circumvent pre-existing immunity. This feature ensures the vaccine can efficiently prime immune responses across diverse populations. Together, these attributes establish SINV as a highly promising platform for developing effective multivalent vaccines against encephalitic alphaviruses.

#### 3.4.2 Virus replicon particles (VRPs) vaccines

VRPs represent an advanced vaccine platform derived from the attenuated VEEV TC-83 strain. These engineered particles retain the viral non-structural proteins (nsP1-4) and cis-acting RNA elements necessary for intracellular RNA replication, but completely lack the native structural genes, which are replaced by a heterologous antigen expression cassette. To optimize immunogenicity, the antigen is placed downstream of the alphavirus 26S subgenomic promoter, and its translation is often enhanced by IRES. A key refinement involves deleting the furin-cleavage site within the glycoprotein gene; this prevents improper processing, helps maintain the native antigen conformation, and is crucial for preserving critical neutralizing epitopes.^64^^,^^65^

VRPs production relies on a bipartite helper system to ensure a single round of infection. The replicon RNA encodes the antigen of interest, while separate helper RNAs supply the structural proteins in trans. This configuration results in the production of non-replicating particles that can enter cells and drive high-level antigen expression through saRNA, but cannot generate new infectious virions, thereby eliminating the risks of environmental release. The process also concurrently activates innate immune sensors such as RIG-I, stimulating a robust interferon response that enhances subsequent adaptive immunity.

Preclinical studies have demonstrated the efficacy of this platform. Reed et al.^64^ developed monovalent and trivalent WEVEE-VRP vaccines (WEVEE-VRP) expressing the glycoproteins of VEEV, WEEV, and EEEV. These candidates elicited potent NAbs in NHPs, with seroconversion rates exceeding 90% for all targeted viruses, and showed no evidence of antigenic interference in the trivalent formulation. Further supporting this approach, Burke et al.^65^ demonstrated that two doses of the trivalent WEVEE-VRP vaccine, administered via various routes, generated high NAb titers and provided complete protection in macaques against lethal aerosol challenge with WEEV. Notably, intradermal delivery induced strong mucosal immunity in the respiratory tract, suggesting a potential to block infection at the primary site of entry.

An alternative strategy to enhance safety involves creating replication-defective vaccines through the deletion of essential replication genes. Zhang et al.^66^ constructed VEEV-ΔnsP4, a candidate with a complete deletion of the RdRp encoding nsP4 gene. A single dose of VEEV-ΔnsP4 was safe in both immunocompetent and immunodeficient mouse models, causing no detectable viremia or disease, yet conferred complete protection against lethal challenge with wild-type VEEV, underscoring its favorable safety and efficacy profile.

Despite their promising immunogenicity, VRP vaccines face significant manufacturing challenges. They are inherently thermolabile, losing most infectivity within 48 hours at 37°C, and downstream purification is complex, often reducing yields to approximately 40% due to the intricate nature of the glycoproteins. Strict quality control is required to remove contaminating helper RNA to levels below 0.1%. Future innovations, such as split-helper systems to minimize recombination and advanced lyophilization techniques to improve thermostability, are essential to overcome these bottlenecks and enable practical deployment.

#### 3.4.3 Recombinant live vector vaccine based on equine herpesvirus (EHV)

The equine herpesvirus-1 (EHV-1) platform utilizes a large double-stranded DNA virus that can be attenuated through specific gene deletions while maintaining high transduction efficiency in mammalian cells. Its substantial packaging capacity, accommodating over 30 kb of foreign DNA, provides a distinct advantage for delivering complex multivalent antigen cassettes that exceed the limits of smaller viral vectors like adenovirus or adeno-associated virus.^67^

To evaluate this platform, Rosas et al.^68^ constructed rH-VEEV, a recombinant EHV-1 expressing the structural polyprotein (E3-E2-6K-E1) of VEEV TrD. In vaccinated mice, this candidate provided complete protection against lethal VEEV challenge, even in the absence of detectable traditional NAbs. Protection correlated instead with strong VEEV-specific IgG/IgG1 antibody responses and CD8⁺ T cells activation, suggesting that mechanisms such as cellular immunity or antibody-dependent cellular cytotoxicity (ADCC) may mediate protection. Regarding safety, while wild-type EHV-1 establishes latency in equine hosts, the recombinant vector has shown no neurotropism or evidence of reactivation from latency in murine or primate models. Clinical monitoring to date has detected no vector shedding over extended periods, though theoretical risks warrant long-term safety surveillance.

#### 3.4.4 Recombinant viral vector vaccine based on modified vaccinia Ankara (MVA)

The MVA vector is a highly attenuated, replication-deficient poxvirus strain with a large genome (approximately 180 kb), enabling the stable insertion and co-expression of multiple heterologous antigens without significant competitive interference.^69^

Hu et al.^70^ established proof of concept for this platform by generating monovalent MVA vaccines against VEEV, WEEV, and EEEV, as well as a trivalent candidate (MVA-BN-WEV). In mice, a two-dose regimen of these vaccines elicited high titers of NAbs (geometric mean NAb titers/GMT > 1∶160) and conferred complete protection against lethal intracranial challenge with the respective viruses. These promising preclinical results supported advancement to human trials. A Phase I clinical trial (NCT04131595) demonstrated that a two-dose schedule of the trivalent MVA-BN-WEV vaccine was safe and highly immunogenic, inducing NAbs in 100% of participants against WEEV and VEEV, and in 92.9% against EEEV. These antibody responses remained detectable in most subjects six months post-vaccination. Robust T-cell responses were also observed in 85% of recipients, supporting the continued clinical development of this candidate.^71^^,^^72^

#### 3.4.5 Recombinant adenovirus vector vaccines

Recombinant adenovirus vectors, notably those based on human adenovirus serotype 5 (Ad5), are well-established vaccine platforms. These replication-defective vectors, typically engineered by deleting the E1 and E3 genes, efficiently transduce antigen-presenting cells such as dendritic cells and macrophages. This property underlies their ability to stimulate potent, Th1-biased immune responses characterized by robust interferon-γ⁺ CD8⁺ T-cell activation and high-titer NAb production. The platform's translational potential is evidenced by the regulatory approval of an inhaled Ad5-vectored COVID-19 vaccine in China, confirming its applicability against respiratory pathogens.^73^

Several Ad5-vectored candidates have been developed against encephalitic alphaviruses. Phillpotts et al.^74^ constructed RAd-VEEV#3, an Ad5 vector expressing the VEEV structural polyprotein (E3–E2–6 K). Intranasal immunization in mice and NHPs elicited high-titer NAbs and VEEV-specific CD8⁺ T cells, conferring 90% protection against lethal aerosol VEEV challenge.

For WEEV, Barabe et al.^75^ developed an Ad5 vector expressing the full E3-E2-6K-E1 polyprotein. A two-dose intramuscular regimen in mice provided complete homologous protection. Adoptive transfer studies further revealed that immune serum could confer partial, FcγR-dependent cross-protection, indicating a role for non-NAbs. The potential for broad protection was further supported by Wu et al.^76^ who demonstrated that a single dose of an Ad5-WEEV vaccine protected mice against antigenically distinct WEEV strains, an effect associated with broadly neutralizing antibodies targeting conserved epitopes in E2 domain III.

The adenovirus platform offers distinct advantages, including a rapid onset of immunity and compatibility with mucosal delivery routes like intranasal immunization, which is critical for blocking respiratory infection at the point of entry. Its inherent ability to infect dendritic cells promotes efficient antigen presentation and T-cell priming. From a manufacturing perspective, the platform is highly scalable, supporting the production of very high viral particle titers.

However, significant challenges remain for widespread deployment. Pre-existing immunity to Ad5, which has a global seroprevalence of 30%–90%, can significantly reduce immunogenicity, particularly for humoral responses, by accelerating the clearance of the vector clearance. This limitation motivates the exploration of alternative strategies, including the use of rare human serotypes (e.g., Ad26, Ad35) or non-human adenoviruses, as well as heterologous prime-boost regimens. Additionally, the robust innate immune activation triggered by TLR9 recognition of viral DNA can lead to transient inflammatory reactions, although clinical data from inhaled Ad5 vaccines have generally shown acceptable safety profiles.

Future development efforts are focused on overcoming these hurdles. Key strategies include engineering chimeric Ad5 vectors with modified hexon proteins to evade pre-existing NAbs, and leveraging rare serotypes with minimal human seroprevalence. The incorporation of molecular adjuvants, such as CD40L, is also being explored to enhance immunogenicity and mitigate post-challenge immunopathology.^77^^,^^78^

#### 3.4.6 Insect-specific Eilat virus (EILV) chimeric vaccine

The EILV represents a novel and highly safe vaccine platform due to its unique host restriction. As an insect-specific alphavirus, EILV is inherently incapable of productive replication in mammalian cells owing to defects in both cell entry and intracellular RNA synthesis. This biological confinement is underscored by safety studies showing no pathogenicity in neonatal mice even after direct intracranial inoculation, along with an absence of detectable innate immune activation. Its biosafety profile enables the construction of chimeric vaccines, such as EILV-EEEV and EILV-WEEV, in which the EILV non-structural protein genes are paired with the structural genes of a pathogenic encephalitic alphavirus. A single subcutaneous dose of these chimeras in adult mice elicits high-titer NAbs and a robust memory CD8⁺ T-cell response, achieving complete protection against lethal viral challenge.^79^ The primary limitations of this promising platform are the current reliance on insect cell culture systems, which requires optimization for industrial-scale production, and the need to validate its immunogenicity in older, more vulnerable populations.

In summary, the field of recombinant vector vaccines for encephalitic alphaviruses encompasses a diverse array of technological solutions. The field has evolved from conventional vectors like SINV and Ad5 to innovative systems like the insect-restricted EILV. Among the current leaders, trivalent MVA-WEV offers broad coverage, while VRP vaccines are notable for their rapid efficacy. The EILV chimera platform, meanwhile, represents a significant advance in biological safety. Persistent challenges common to these platforms include overcoming pre-existing immunity in human populations, achieving cost-effective manufacturing at scale, and ensuring robust immunogenicity in the elderly.

### 3.5 Subunit and virus-like particle vaccines

Subunit vaccines and virus-like particles (VLPs) constitute a highly safe and immunogenic vaccination strategy, as they utilize specific, purified pathogen components rather than whole viruses. Recombinant protein vaccines employ purified antigens to elicit immunity, though their often limited immunogenicity necessitates the use of potent adjuvants. In contrast, VLPs self-assemble into non-infectious structures that mimic native virions, combining an excellent safety profile with enhanced immunostimulatory properties.

#### 3.5.1 Recombinant protein vaccines

The effectiveness of recombinant protein vaccines is highly dependent on both the structural authenticity of the antigen and the adjuvant system employed. For instance, Das et al.^80^ immunized mice with *E. coli*-expressed WEEV E1 protein. Although three immunizations generated E1-specific antibodies, they conferred only limited protection against a homologous aerosol challenge and no protection against a heterologous strain, underscoring the importance of the native antigen conformation for inducing potent NAbs. A similar outcome was observed with recombinant E2 vaccines, which provided only partial protection.^81^

Conversely, innovative adjuvant systems can sometimes elicit protection through non-canonical immune pathways. Rico et al.^82^ developed a complex combining VEEV/WEEV E1 antigens with cationic liposomes and immunostimulatory nucleic acids (dsRNA + CpG DNA). Remarkably, two subcutaneous doses in mice generated non-NAbs that provided complete cross-protection against lethal challenge with VEEV, WEEV, and EEEV. This surprising result suggests that synergistic activation of innate immune pathways (e.g., via TLR3/9) can compensate for the absence of traditional NAbs, offering a novel protective mechanism.

Further refining this approach, Huang et al. engineered a sophisticated liposomal adjuvant system (designated CPQ) to present His-tagged consensus antigens from VEEV and EEEV. This formulation significantly enhanced antigen-specific IgG titers compared to antigens delivered alone, demonstrating the potential of sophisticated adjuvant technology. However, the neutralization breadth achieved remained suboptimal, and the vaccine's efficacy awaits validation in animal challenge models.^83^

In summary, recombinant protein vaccines offer advantages in scalable production and safety. Their principal challenges lie in preserving native antigen conformation during manufacturing and formulating effective adjuvant systems to overcome their inherently weaker immunogenicity.

#### 3.5.2 Virus-like particle (VLP) vaccines

VLPs are non-infectious nanoparticles that self-assemble from viral structural proteins, accurately mimicking the native architecture of virions. By presenting conformational antigenic epitopes, VLPs efficiently engage B-cell receptors and are readily internalized by antigen-presenting cells, thereby stimulating robust humoral and cellular immunity without any risk of viral replication.

Considerable progress has been made in developing VLP vaccines against encephalitic alphaviruses. For instance, the trivalent candidate VRC-WEVVLP073-00-VP (developed by NIAID), which incorporates structural proteins from VEEV, WEEV, and EEEV, elicited broad NAbs and conferred complete protection against all three viruses in animal models, validating its potential as a safe and potent multivalent strategy.^83^ This promising approach has advanced to clinical evaluation. A Phase I clinical trial (NCT03879603) demonstrated that both alum-adjuvanted and unadjuvanted formulations were safe and well-tolerated in healthy adults, inducing durable NAbs that persisted for at least six months.^84^ From a manufacturing perspective, Ma et al.^85^ established a scalable production method for WEEV VLPs using a baculovirus-insect cell system. When formulated with AddaVax adjuvant, these VLPs stimulated a balanced Th1/Th2 immune response and high-titer NAbs in mice, leading to complete protection against lethal challenge.

Despite their excellent immunogenic properties, VLP vaccines face specific production challenges. The manufacturing process is inherently complex, requiring the precise assembly of multiple structural proteins into stable particles. While multivalent designs like VRC-WEVVLP073 represent the forefront of development, they must additionally overcome the hurdle of ensuring the correct and stable co-assembly of heterologous proteins from different viruses.

#### 3.5.3 Multiepitope vaccines (MEVs)

Multiepitope vaccines (MEVs) employ a rational design, utilizing computational tools to select immunodominant epitopes while excluding potentially allergenic or immunosuppressive sequences. This approach aims to maximize immune coverage and target conserved regions across viral variants. However, it carries the inherent risk of incomplete protection if the selected epitopes are not efficiently processed or presented *in vivo*.

Exemplifying this strategy, Nguyen et al.^86^ designed an EEEV-MEV candidate through immunoinformatic screening. The construct incorporated three MHC-I epitopes, five MHC-II epitopes, and two B-cell epitopes from EEEV, fused to *Salmonella flagellin* as a built-in TLR5 agonist to enhance immunogenicity. *In silico* analysis confirmed stable binding of the construct to TLR5, and predicted strong IgG/IgM responses with B-cell memory formation. While such computational design enables rapid candidate generation, the predicted efficacy of the EEEV-MEV requires thorough experimental validation. Key questions remain regarding actual epitope processing, T-cell priming efficiency, and cross-strain protection breadth. Furthermore, the safety of using flagellin as an adjuvant must be carefully assessed, due to its potential to induce inflammatory reactions.

#### 3.5.4 Self-assembling nanoparticle vaccines

Beyond the established platforms, self-assembling nanoparticle vaccines represent a transformative strategy that merges the precise antigen display of VLPs with programmable subunit design. This approach employs synthetic biology to engineer protein subunits that spontaneously assemble into nanostructures, presenting viral antigens in a highly ordered, repetitive array.^87^^,^^88^ This dense, multivalent display can most effectively activate B cell receptors, promote robust germinal center responses, and induce the production of potent antibodies that often possess broad neutralizing activity.^89^

Proof-of-concept for this platform against other pathogens is promising. For example, a ferritin-based nanoparticle displaying the Nipah virus G glycoprotein head domain elicited significantly broader and more potent NAbs against multiple henipaviruses in mice, compared to its soluble counterpart.^90^ In another innovative approach, a mRNA vaccine was designed to instruct host cells to produce proteins that self-assemble *in vivo* into nanoparticles displaying the SARS-CoV-2 receptor-binding domain. This strategy combined mRNA delivery with nanostructured presentation, yielding antibody 5 to 28 times higher than those from a standard spike mRNA vaccine in mice.^91^

Key technological advantages of this platform include high design flexibility, precise control over antigen architecture and valency, and potential for improved stability. Novel manufacturing methods, such as polymer-based nanoparticles capable of room-temperature assembly in water, offer promising avenues to further simplify production and facilitate deployment by reducing cold-chain dependence.^92^

For encephalitic alphaviruses, the self-assembling nanoparticle vaccine platform holds significant yet largely unexplored potential, with unique design considerations and comparative advantages that address key limitations of conventional vaccine strategies. The core of its mechanism is the ability to drive B cell receptor clustering through the display of multivalent epitopes, which is a hallmark of its superior immunogenicity compared to soluble antigen-based vaccines.^58^ Guided by rational design principles, the primary neutralizing targets, the E1 and E2 glycoproteins, could be engineered as fusion proteins to form core nanoparticles or be displayed on inert protein scaffolds (e.g., ferritin or I53-50). This modular design not only provides a high degree of flexibility, but more importantly, enables the targeted presentation of structurally conserved epitopes in VEEV, WEEV, and EEEV strains (such as the E1 fusion loop or E2 domain B region), thereby directly addressing the core challenge in developing a single broad-spectrum cross-protective vaccine.^93^^,^^94^ However, critical experimental gaps must be addressed. Current validation studies have critical limitations: (1) Most studies focus on a single or a few viral strains, lacking systematic cross-strain assessments of immunogenicity and protective efficacy against major encephalitic viruses (VEEV, WEEV, and EEEV)^95^; (2) High-resolution structural data necessary for rational design are lacking, and the characterization of E1/E2 broadly neutralizing antibody complexes and critical antigenic conformations (such as the prefusion complex) remains incomplete^96^; (3) Basic characterization of recombinant antigens (such as testing their integrity and specificity via immunoblotting) is often overlooked or inadequately reported; (4) Preclinical studies lack depth, with most vaccine candidates tested only in rodents, and an urgent need for validation in non-human primates and human-relevant models; (5) The systemic optimization and evaluation of nanoparticle platforms' *in vivo* pharmacokinetics of nanoparticle platforms (lymph node targeting efficiency, immune durability) are still insufficient.^97^ Moreover, although challenges remain in manufacturing complex heterogeneous protein assemblies, this platform has established its position as a key direction in future research on broad-spectrum alphavirus encephalitis virus vaccines due to its ability to elicit exceptional immune responses, combined with its modular design.

In sum, the subunit vaccine field is evolving through multiple advanced strategies, including structural antigen engineering, innovative adjuvant systems, and rational designs like VLPs and MEVs. Currently, VLP platforms lead in clinical translation due to their favorable balance of safety and efficacy, exemplified by multivalent candidates such as VRC-WEVVLP073. Recombinant protein vaccines require breakthroughs in antigen design to overcome their inherent weak immunogenicity. Computational MEVs remain high-risk and high‑reward candidates dependent on experimental validation. Critical future priorities for all subunit platforms include developing thermostable formulations for practical deployment in tropical regions and reducing production costs to ensure global accessibility.

## 4 Perspectives and future directions

The continued spread of encephalitic alphaviruses, propelled by global travel and climate-driven expansion of vector habitats, underscores the urgent need for effective vaccines against VEEV, WEEV, and EEEV. While traditional vaccines like inactivated formulations have demonstrated utility, their limitations in protective breadth and durability have accelerated the development of next-generation platforms. Two core challenges dominate the field: achieving broad cross-protection and ensuring durable, safe immunity.^98^^,^^99^

The recent approval of two chikungunya vaccines—the live-attenuated IXCHIQ® and the virus-like particle-based VIMKUNYA™—has provided valuable technological and regulatory precedents. These successes highlight several promising avenues for encephalitic alphaviruses. mRNA vaccines offer rapid design and potent immunogenicity, though requiring improvements in thermostability; VLP and nanoparticle vaccines, which mimic native virion architecture to enhance immunogenicity without replication risks, albeit with manufacturing complexities; and recombinant viral vectors, which enable multivalent antigen expression and single-dose efficacy but must overcome pre-existing immunity and specific safety considerations.^93^

Addressing the core challenges requires strategic innovation. For LAVs, multiple stable mutations can be introduced through reverse genetics technology to achieve rational attenuation, thereby improving the balance between immunogenicity and safety while minimizing the risk of reversion mutations. The approach systematically transforms vaccine development from “empirical attenuation” to “precision design” by accurately introducing multiple, stable synergistic mutations. This approach involves the strategic overlay of various attenuation mutations (such as point mutations and codon deoptimization) in key regions of the viral genome (such as the replicase or structural protein genes) to achieve an extremely low probability of virulence reversion, thereby significantly enhancing vaccine safety. Additionally, intact protective antigenic epitopes are deliberately preserved to ensure the induction of a robust and balanced immune response. This strategy has been successfully validated in the context of vaccine development for influenza and Zika viruses, thereby marking the entry of LAVs into an era of predictable, controllable “engineered vaccines” and providing a critical pathway for developing safer, more effective broad-spectrum vaccines.^100^ For nucleic acid and subunit vaccines, enhancing their durability requires the use of advanced adjuvants, heterologous prime-boost immunization regimens, and nanoparticle-based platforms to promote robust germinal center immune responses.^101^ Through multidimensional synergistic strategies, the durability of nucleic acid vaccines and subunit vaccines can be further improved. These technologies include: advanced adjuvant systems that create a microenvironment favorable for germinal center formation by directional activation of innate immunity; heterologous prime-boost immunization strategies that progressively stimulate a broader range of B and T cell memory through sequential timing; and antigen delivery technologies (such as nanoparticles) that efficiently activate B cells through multivalent, organized structures, strongly driving their differentiation into long-lived plasma cells and memory B cells. The synergistic effect of these three approaches collectively facilitates the critical shift of the immune response from passive induction to active design.^102^

Advances in structural vaccinology are providing a foundation for rational antigen design, a capability that can powerfully synergize with these novel platforms. For instance, the recent elucidation of VEEV’s receptor-binding mechanism with LDLRAD3 offers a blueprint for targeting conserved epitopes across alphaviruses.^103^^,^^104^ Such insights directly inform platform engineering, as demonstrated in alphavirus VRPs where deletion of the furin cleavage site help stabilize key neutralizing epitopes. For self-assembling nanoparticles, these structural data could guide the precise engineering of E1/E2 glycoproteins, potentially enabling a single vaccine with broad cross-protective efficacy against multiple encephalitic alphaviruses.^105^

## 5 Translational challenges and the role of animal models

Nevertheless, translational research still faces significant challenges. The process of translating findings from preclinical research to clinical application is often fraught with difficulties, primarily due to the limitations of animal models and the absence of defined immune protection-related indicators, which have yet to be clearly defined. Although this model has been widely used in initial proof-of-concept studies, it still has limitations in simulating the neuroinvasive processes, cellular tropism, and immunopathological mechanisms of human encephalitis, often failing to recapitulate the complex neuroinvasive disease observed in humans. Non-human primate models offer enhanced fidelity; however, they are also significantly more resource-intensive. Furthermore, data pertaining to NHPs, particularly with regard to EEEV, is rather limited.^106^, ^107^, ^108^, ^109^ Crucially, the low incidence of human disease makes it difficult to conduct large efficacy trials and establish clear clinical correlates of protection (e.g., a specific NAb titer that reliably predicts immunity). This uncertainty complicates decision-making and hinders the clinical advancement of promising candidates. Manufacturing bottlenecks, particularly for innovative platforms like self-assembling nanoparticles and the insect-restricted EILV system, further complicate development.

Therefore, a synergistic strategy that integrates computational immunogen design, cross-protective platform technologies, and thermostable formulations is essential. Future efforts should prioritize: (1) developing modular antigen platforms that leverage structural insights to target conserved epitopes across VEEV, WEEV, and EEEV; (2) establishing global immunogenomics initiatives to define T-cell correlates of protection across diverse populations; (3) Combining geospatial vector forecasting with heat-stable vaccine formulations, which remain stable for over 12 months at temperatures between 2 and 8°C, represents a significant advancement in the field. The implementation of this model should target single-dose or simplified dosing schedules (e.g., 1–2 doses) to enhance vaccination coverage. In addition, controlling the production cost per dose to a few dollars (for example, targeting a range of $2–$5) is crucial for achieving vaccine accessibility in resource-limited regions. Integrating self-assembling nanoparticle technology to efficiently present rationally designed antigens in a multivalent array format is expected to provide a blueprint for developing the next-generation vaccines with unprecedented breadth and potency.

## CRediT authorship contribution statement

**Xiaojing Yang:** Writing – original draft. **Yuying Ning:** Writing – original draft. **Chengnan Xu:** Writing – review & editing, Funding acquisition. **Qianqian Zhang:** Writing – review & editing. **Yangchao Dong:** Writing – original draft. **Yuan Wang:** Writing – review & editing, Funding acquisition. **Fanglin Zhang:** Writing – review & editing, Funding acquisition. **Yingfeng Lei:** Writing – review & editing, Supervision. **Wei Ye:** Writing – review & editing, Funding acquisition.

## Informed consent

Not applicable.

## Organ donation

Not applicable.

## Ethical statement

Not applicable.

## Data availability statement

Data sharing is not applicable to this article as no datasets were generated or analysed.

## Animal treatment

Not applicable.

## Generative AI

We confirm that AI-assisted technologies were only used for language polishing, and all scientific content, data, and interpretations were created and verified by the authors.

## Funding

This work was supported in part by the 10.13039/501100013290National Key Research and Development Program of China (No. 2022YFC2604200), 10.13039/501100001809National Natural Science Foundation of China (No. 82072268), 10.13039/501100007547Fourth Military Medical University supporting grants (Nos. 2022ZZXM044 and 2021JSTS10), and Youth Innovation Team Project of Shaanxi Provincial Department of Education (23JP194). The funders had no role in the study design, data collection, analysis, publication decision, or manuscript preparation.

## Declaration of competing interest

The authors declare that they have no known competing financial interests or personal relationships that could have appeared to influence the work reported in this paper.
